# Ionic Liquid-Doped Gel Polymer Electrolyte for Flexible Lithium-Ion Polymer Batteries

**DOI:** 10.3390/ma8052735

**Published:** 2015-05-20

**Authors:** Ruisi Zhang, Yuanfen Chen, Reza Montazami

**Affiliations:** 1 Department of Mechanical Engineering, Iowa State University, Ames, IA 50011, USA; E-Mails: ruisizhang@yahoo.com (R.Z.); yuanfenc@iastate.edu (Y.C.); 2 Center for Advanced Host Defense Immunobiotics and Translational Comparative Medicine, Iowa State University, Ames, IA 50011, USA

**Keywords:** ionic liquid, gel polymer electrolyte, flexible electronics, lithium-ion polymer battery

## Abstract

Application of gel polymer electrolytes (GPE) in lithium-ion polymer batteries can address many shortcomings associated with liquid electrolyte lithium-ion batteries. Due to their physical structure, GPEs exhibit lower ion conductivity compared to their liquid counterparts. In this work, we have investigated and report improved ion conductivity in GPEs doped with ionic liquid. Samples containing ionic liquid at a variety of volume percentages (vol %) were characterized for their electrochemical and ionic properties. It is concluded that excess ionic liquid can damage internal structure of the batteries and result in unwanted electrochemical reactions; however, samples containing 40–50 vol % ionic liquid exhibit superior ionic properties and lower internal resistance compared to those containing less or more ionic liquids.

## 1. Introduction

Li-ion batteries are among the most promising, efficient and common high-energy-density systems for electrochemical energy storage. In recent years application of Li-ion batteries in common electronic devices, and thus demand for more efficient and safer batteries, has increased significantly [1,2,3,4]. Batteries with higher efficiency, superior mechanical properties, and smaller size [5] are needed for handheld electronics to keep up with the rapidly increasing computing power, larger screens and thinner and lighter designs of such devices. In addition, there is increasing need for polymer-based batteries to be incorporated with flexible, soft and micro electronics [6,7,8,9,10,11]. There has also been a significant increase with concerns regarding the issues associated with such batteries. Use of flammable organic solvents as electrolyte, formation of lithium dendrites, and large volume change due to poor structural stability are among the main concerns associated with Li-ion batteries. Use of gel polymer electrolytes (GPEs) has addressed some concerns regarding leakage of liquid electrolytes and the resultant fire hazards; however, charge transfer through GPE doped with organic solvents is not as efficient as that in liquid electrolytes. Also, doping GPE with organic solvents poses some limiting difficulties.

Generally, gel polymer electrolytes’ synthesis is achieved by incorporating an organic electrolyte solution into a polymer matrix with a trapping structure enhanced by carbonate esters [12,13]. Polymer matrices with high chemical stability and strong electron-withdrawing functional groups to induce a net dipole moment are desirable as the polymer host [14]. One polymer commonly used in gel polymer electrolytes is polyvinylidene fluoride (PVdF), containing -C-F functional groups. The PVdF base gel polymer electrolyte membranes attract ions in the organic electrolyte solution due to the electric field at the surface of the PVdF membrane. Because of the semi-crystalline structure of PVdF, parts of the attracted ions are drafted into the membrane when the rest of the ions stay at the surface [15,16,17,18,19,20]. Thus, a gel polymer electrolyte membrane with fully interconnected open micropores, *i.e.*, higher interfacial surface area, enhances ion storage and mobility [17,21,22,23,24,25,26,27,28].

Compared to liquid electrolytes, gel polymer electrolytes have several advantages, such as faster charging/discharging, and potentially higher power density [29,30,31]. However, ion permeability of gel polymer electrolytes is orders of magnitude lower than that of liquid electrolytes, mainly because of the polymeric structure which limits the ion mobility [1,14].

Room temperature ionic liquids have been used as substitutes for organic electrolytes to increase ion mobility throughout the electrolyte and also to eliminate hazards associated with organic electrolytes. Application of ionic liquids in lithium-ion batteries has been the focus of several studies in the recent years. Fernicaola *et al.* incorporated ionic liquids in an organic electrolyte solution to increase the ionic conductivity and stabilize the lithium ions carried on the surface of PVdF based membrane [32]. In one study Egashira *et al.* have shown than the ion mobility through the gel electrolyte containing ionic liquids depends on the miscibility of polymer component in the ionic liquid. It was shown that, for example, gel electrolytes containing hexyltrimethylammonium bis(trifluoromethane sulfone)imide ionic liquid exhibits high lithium ion permeability, whereas no obvious lithium ion mobility was detected through a gel electrolyte containing 1-ethylk-3-methyl imidazolium bis(trifluoromethane sulfone)imide ionic liquid [33]. In other studies it was demonstrated that the ion permeability of the gel electrolyte could be improved by the addition of carbonate esters. Carbonate esters play the role of ion dissociation enhancer and improve ion mobility because of their relatively high dielectric constants (ε). Ethylene carbonate (ε = 89.78 at 40 °C) and propylene carbonate (ε = 64.93 at 25 °C) are among the most common carbonate esters used in lithium-ion polymer batteries. They both have excellent thermal stability and a boiling point of above 240 °C [34]. Ye *et al.* doped the gel electrolyte by a small amount of ethylene carbonate and observed a significant increase in lithium ion transport through the gel electrolyte [35]. Sirisopanaporn *et al.* demonstrated higher ion permeability and interfacial stability by the addition of small amounts of ethylene carbonate and propylene carbonate to the gel electrolyte [36]. A vapor-free lithium-ion polymer battery with high discharge performance based on lithium salt dissolved in ionic liquid and ultra-high molecular weight ionic liquid polymer was reported by Sato *et al.*; it was demonstrated that the discharge performance is higher than that of a conventional lithium polymer batteries [37].

In this work we attempt to fill the gap between efficient systems based on organic solvents and safe and reliable systems based on ionic liquids. We have investigated GPEs doped with a mixture of organic electrolyte and ionic liquid at different ratios, in the presence of carbonate esters, to enhance ion permeability and electrochemical properties of the GPEs. We have characterized GPEs for their electronic properties and have also investigated their performance in lithium-ion polymer batteries as a function of ionic liquid content.

## 2. Materials and Methods

### 2.1. Materials

Copper foil single-side coated by 0.1 mm of composite graphite anode and aluminum foil single-side coated by 0.1 mm of lithium manganese oxide (LiMn_2_O_4_) cathode were purchased from MTI corporation (Richmond, CA, USA) and used as received. *N*-Methyl-2-pyrrolidone (NMP), ethylene carbonate (EC), propylene carbonate (PC), lithium hexafluorophosphate (LiPF_6_), polyvinylidene fluoride (PVdF), and 1-Ethyl-3-methylimidazolium triluoromethanesufonate (EMI-Tf) were purchased from Sigma-Aldrich (St. Louis, MO, USA) and used as received. LiPF_6_ was selected as the lithium salt due to its high conductivity in carbonates solvent mixtures, also due to its ability to prevent aluminum corrosion at the cathode aluminum current collector by forming a passivation layer. EMI-Tf was selected because of its high ionic conductivity (10^−2^ S/cm) and wide electrochemical window.

### 2.2. Electrolyte

Electrolyte solutions with different compositions were prepared by dissolving 1 M of LiPF_6_ in a solvent consisting of EC, PC and EMI-Tf at different ratios. EC, PC and EMI-Tf were mixed at desired ratios (see Table 1) and stirred for at least 2 h; lithium hexafluorophosphate was then added to the solvent under an inert gas environment to achieve 1 M concentration, and stirred for 24 h.

**Table 1:** Gel polymer electrolyte (GPE) composition. Volume percentage (vol%) of EMI-Tf is also used as samples’ names.

Compound	GPE Composition (vol%)							
EMI-Tf	0%	25%	30%	40%	50%	60%	75%	100%
EC	50%	37.5%	35%	30%	25%	20%	12.5%	0%
PC	50%	37.5%	35%	30%	25%	20%	12.5%	0%

### 2.3. Membrane Synthesis and Activation

The membrane was synthesized by first preparing a carbonate ester mixture. A 1:1 weight ratio mixture of EC and PC was heated to 80 °C to achieve complete dissolution. The resultant clear carbonate ester solution was mixed with PVdF and 1-Methyl-2-pyrrolidone at 10:4:11 weight ratio. The mixture was then heated to 110 °C and stirred on a magnetic stirrer until a clear solution was obtained with a relatively high viscosity. The solution was then casted on a glass template and dried under vacuum at 80 °C for 2 h to form membranes. The membranes were then soaked in a 10% ethanol aqueous solution overnight. Pale yellow membranes with 50 μm thickness were obtained and cut into 22 mm × 22 mm squares and stored under ambient conditions. Membranes were then activated by full exposure (immersion) to electrolyte solution for 24 h. Electrolyte content was measured as the weight percentage (wt %) of dry weight of the membrane, and calculated from Equation (1).

(1) $$W_{e}(\%)=\frac{W_{f}-W_{d}}{W_{f}}\times 100$$

where *W_e_* (%) is the weight-percent of the electrolyte; and, *W_d_* and *W_f_* are the weights of dry and doped membranes, respectably. All membranes reached an approximate 35 wt % electrolyte content which is the typical upper limit saturation.

**Figure 1:**
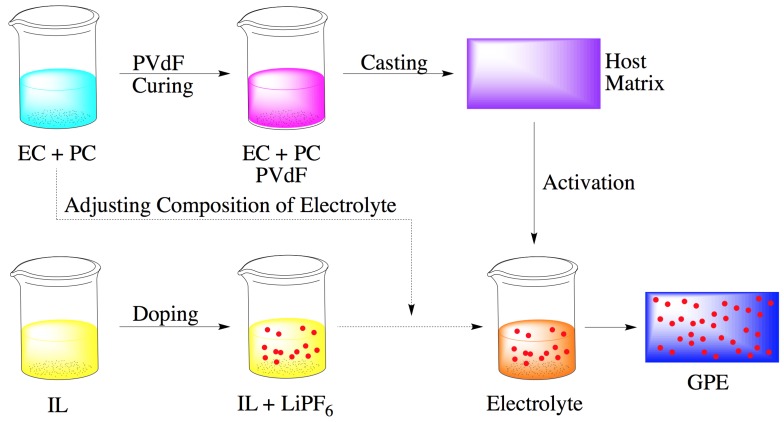
Schematic of the process for fabrication and activation of GPEs.

Schematic of the fabrication and activation processes are presented in Figure 1.

### 2.4. Full Cell Assembly

**Figure 2:**
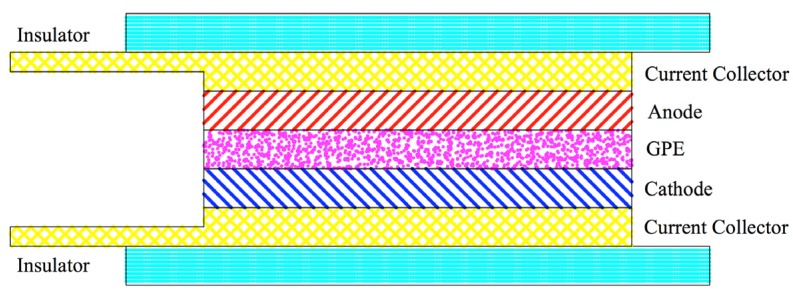
Schematic of test cell structure.

**Figure 3:**
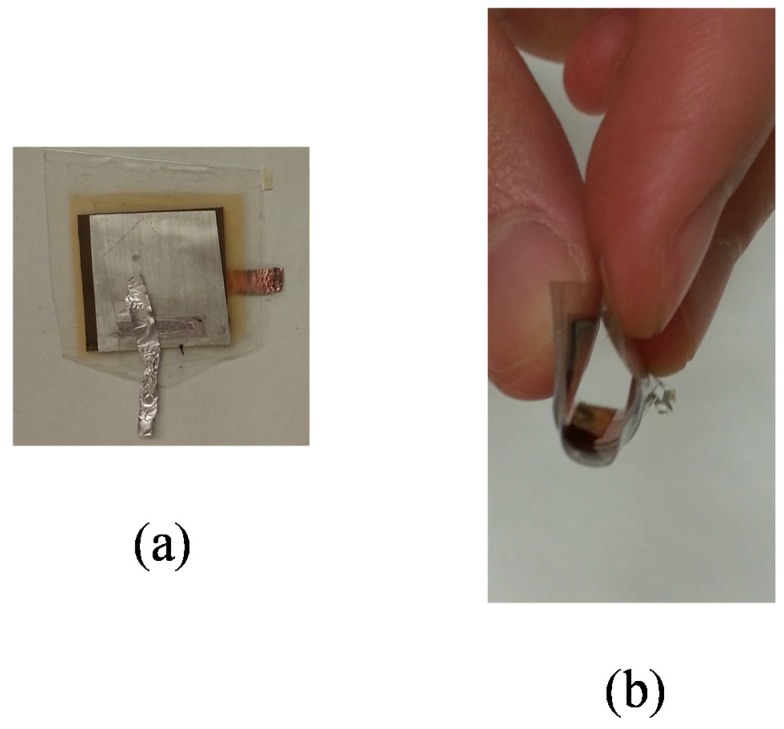
(**a**) Photograph of a sample battery structure; (**b**) bending of the battery under mechanical stress.

The anode material of Graphite was casted on the surface of copper foil as the current collector; the cathode material of LiMn_2_O_4_ was casted on the surface of aluminum foil as the current collector. Schematic of the test cell is presented in Figure 2, with GPE located in between the cathode and anode. Cathode and anode are exactly 20 mm × 20 mm; but GPE film is larger than cathode and anode so the cell would not be shorted. The surface of protection cover that faces inside of the cell is adhesive, which helped airtight enclosure of the whole system. The actual model is displayed in Figure 3a; the flexibility of the cell is shown in Figure 3b.

### 2.5. Measurement

A VersaSTAT-4 potentiostat (Princeton Applied Research, Oak Ridge, TN, USA) was used for electrochemical and impedance spectroscopy studies of the gel polymer electrolyte membranes. For these studies GPE membranes were secured between two steel-disks electrodes of 200 μm thickness and 15.5 mm diameter, and two pieces of adhesive plastic were used as a pouch to seal and hold each sample. The impedance spectroscopy of full cells studies were carried at a frequency between of 1.0 × 10^5^ and 0.1 Hz and a potential difference (ΔV) of 10 mV, after completion of 10 charging/discharging cycles. In each sample, the membrane was cut slightly larger than the electrodes to prevent short-circuit. Charging-discharging tests were carried out with a computer controlled BST8-MA battery analyzer (MTI corporation), between 0.5 and 5 V with a constant current of 5 mA.

## 3. Results and Discussion

### 3.1. Ionic Conductivity

**Figure 4:**
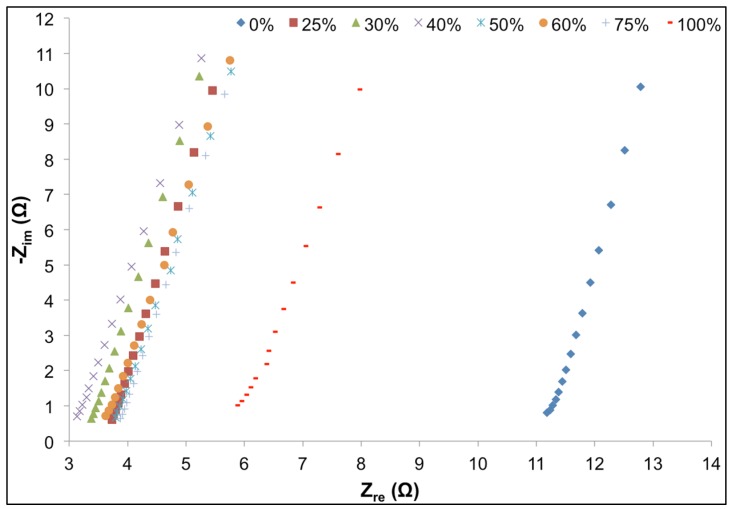
Nyquist plots of systems consisting of GPEs with different volume percent ionic liquid at high frequency.

**Figure 5:**
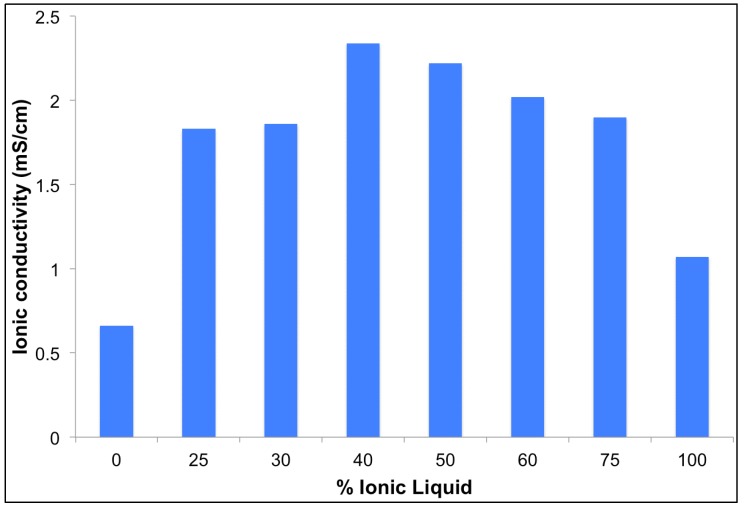
Ionic conductivity of GPEs as a function of IL content.

Ion conductivity of GPEs was studied by AC impedance spectroscopy. GPEs doped with electrolytes of different composition were secured between two steel disks and studied at a high frequency range (10–100 kHz). As presented in Figure 4, the Nyquist plot exhibited approximately vertical lines, suggesting nearly pure resistive behavior at high frequencies, for all GPE samples. Here, the effect of the imaginary part of the impendence can be neglected and the system can be considered as a pure resistor with minimum dependence on frequency. The internal resistance values of the bulk electrolytes can be induced from the intercept of the extended impedance plots with the *x*-axis (*Z_re_*) (see Figure 5). Ionic conductivity of GPEs can be calculated using internal resistance, thickness and surface area of the GPEs. The ionic conductivity σ for each GPE sample was calculated using Equation (2):

(2) $$\text{σ}=\frac{t}{RA}$$

where *t* is the thickness of each sample, *R* is the internal resistance and *A* is the surface area (1.89 cm^2^). Ionic conductivities of the GPE samples are presented in Table 2.

**Table 2:** Characteristic properties of GPEs as a function of IL content.

*A* (cm^2^)	*IL* (vol %)	*R* (Ω)	*t* (cm)	σ (mS·cm^−1^)
1.89	0	11.2379	0.014	0.66
1.89	25	3.7595	0.013	1.83
1.89	30	3.4118	0.012	1.86
1.89	40	3.1770	0.014	2.34
1.89	50	3.8264	0.016	2.22
1.89	60	3.6806	0.014	2.02
1.89	75	3.9111	0.014	1.90
1.89	100	5.9292	0.012	1.07

Samples containing EMI-Tf ionic liquid exhibited ~65% to ~270% improvement on their ion permeability compared to a sample without EMI-TF (100% EC-PC solvent), and samples with only EMI-TF. Specifically, samples with 40 and 50 vol % ionic liquid had the highest ionic conductivity. Interestingly, ion permeability of the GPEs showed an increasing trend as the ionic liquid content increased to ~40% and decreased thereafter, suggesting the significant contribution of EC and PC toward ion permeability of the GPEs; and potentially, formation of ionic double layers at electrodes which retard ion mobility, as observed and reported previously [9]. It is important to note that in this study the ion permeability of PVdF-based host matrix is deduced from the movement of all salt and ionic liquid ions (Li^+^, EMI^+^, Tf^−^, and PF_6_^−^, EMI^+^ and Tf^−^ only when ionic liquid was used) in the GPE. Previous studies [24,38,39,40] have shown that among cations, the EMI^+^ always diffuses faster than smaller Li^+^, and that Li^+^ and anions are more likely to form ion complexes and diffuse together at a slower rate. Also, high ionic conductivity of EMI-Tf (6.4 mS/cm) at room temperature contributes significantly to the ion permeability of the GPE membrane [41,42]. Yet, GPE’s containing EMI-Tf as the only solvent exhibited relatively low ionic conductivity, comparable to that of samples only containing EC-PC solvent.

### 3.2. Interfacial Properties

**Figure 6:**
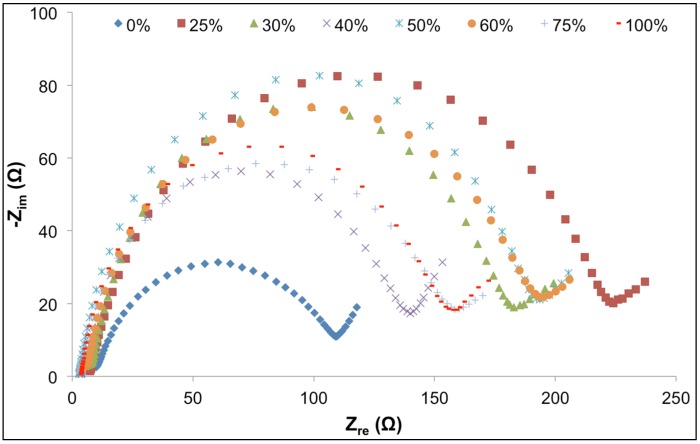
Nyquist plots of Li-ion polymer batteries consisting of GPEs with different volume percent ionic liquid.

The interfacial properties of GPEs were determined by impedance spectroscopy (1.0 × 10^5^ to 0.1 Hz, Δ*V* = 10 mV) in thin-film cell pack configuration, after completing 10 cycles of charging and discharging. As presented in Figure 6, the curves of Nyquist plots for all cells are semicircular, followed by a linear segment, at approximately 45° slope, indicating Warburg impedance.

**Figure 7:**
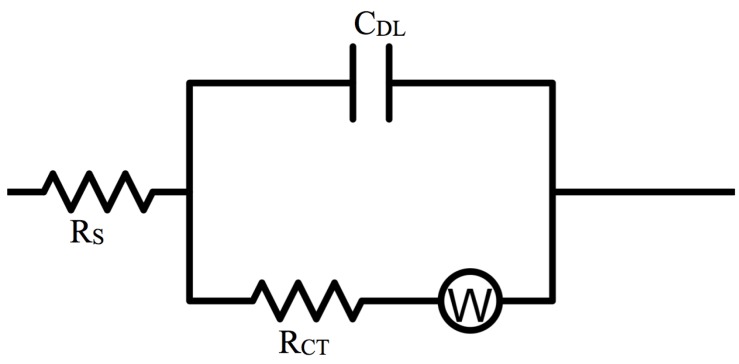
Randles equivalent electrical circuit is used to describe Lithium-ion polymer battery cells.

At high frequencies, close to the origin of the *x*-axis, the electrochemical systems exhibited pure resistance behavior. Intersection of the semicircular plots with the *x*-axis, at high frequency regions, manifests the solution resistance (*R_s_*), as presented in the Randles equivalent electrical circuit (Figure 7). Solution resistance depended on the ion content of the entire system and transportation of ions between anode and cathode; and it is slightly different than ionic conductivity, discussed in the previous section. Solution resistance of samples, listed in Table 3, suggests that the addition of ionic liquids results in reduction of solution resistance, an observation that is in agreement with expected effect of any ion-rich electrolyte, such as ionic liquids. A slight increase in solution resistance was observed for 100% ionic liquid electrolyte that is anticipated to be a result of high concentration of ions in the ionic liquid, limiting mobility of charge.

**Table 3:** Electrical properties of samples as a function of ionic liquid content, in accordance with Randles equivalent electrical circuit.

*ILs* (vol %)	*R_S_* (Ω)	*R_CT_* (Ω)	*C_DL_* (F)	σ_S_
**0**	9.983	98.940	3.99 × 10^−4^	78.814
**25**	7.475	216.578	3.03 × 10^−4^	95.976
**30**	5.517	177.381	4.25 × 10^−4^	99.088
**40**	3.785	136.122	4.43 × 10^−4^	113.52
**50**	2.849	190.677	4.80 × 10^−4^	97.602
**60**	3.968	189.382	4.43 × 10^−4^	95.877
**75**	3.065	155.311	5.36 × 10^−4^	84.32
**100**	2.931	154.235	6.54 × 10^−4^	98.015

**Figure 8:**
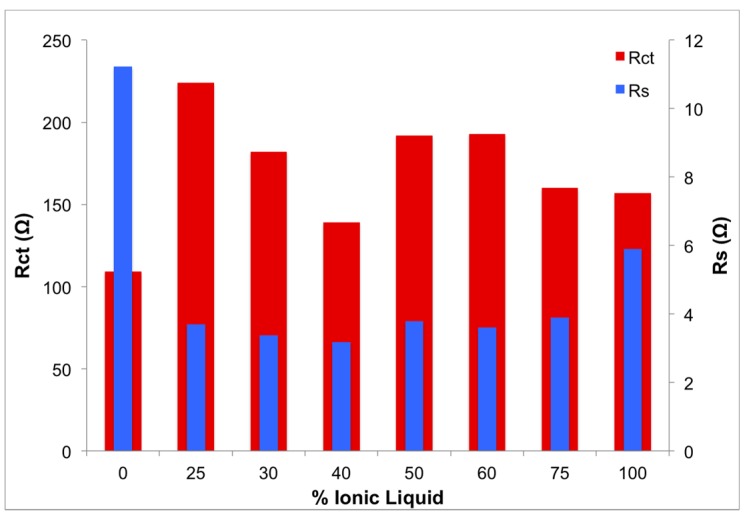
Solution and charge transfer resisstance as a function of ionic liquid content.

The diameters of the semicircles represent charge transfer resistance (*R_CT_*); by assuming the semicircles are associated with parallel combination of charge transfer resistance (*R_CT_*), and double layer capacitance (*C_DL_*) and Warburg impedance (*W*) in series, the system could be described as the equivalent circuit presented in Figure 7 [43,44]; *R_S_* and *R_CT_* data are presented in Figure 8. Double layer capacitance (*C_DL_*) was calculated using *Z_im_* from the Nyquist plots at pure capacitance points (phase angle = π/2) and the corresponding frequency (Equation (3)) [45,46]. Generally, *C_DL_* showed an increasing trend with an increase of ionic liquid content in the GPE, which translates to higher storage capacity, due to increased concentration of ions and resultant decreased solution resistance.

(3) $$C_{DL}=\frac{2\pi }{f\times Z_{im}}$$

**Figure 9:**
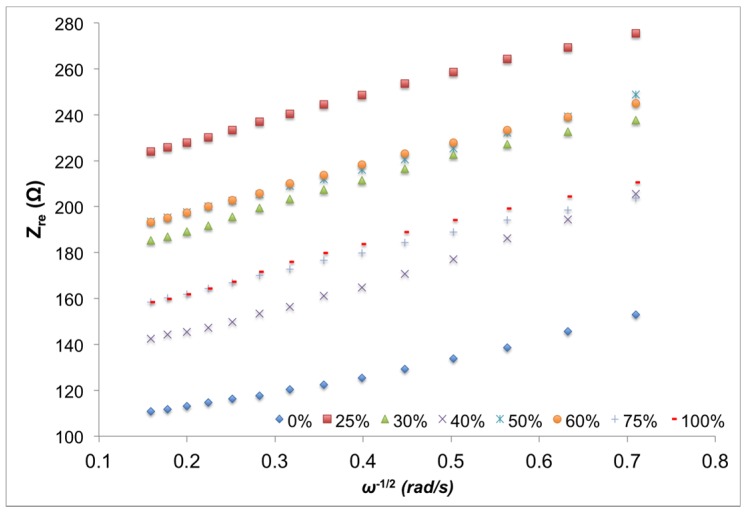
Real impedance verses the inverse of the square root of radial frequency at low frequency domain (63–3 Hz).

Warburg coefficient (σ_S_) is deduced from the slope of the plot of square root of radial frequency at low frequency domain *vs.* the real impedance values. The corresponding plot is presented in Figure 9. All electrical properties of the samples, in accordance with Randles equivalent electrical circuit, are summarized in Table 3.

### 3.3. Battery Performance

Galvan static charging and discharging were used to evaluate to performance of lithium-ion polymer batteries *containing GPE of different ionic liquid content.* Each cycle included constant current (0.5 A) discharging followed by an 8 min rest period, then constant current and constant voltage charging.

**Figure 10:**
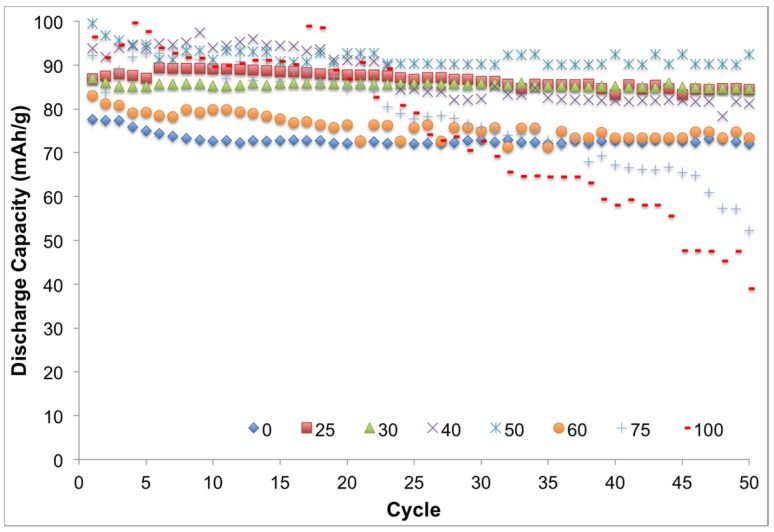
Consecutive cycling behavior of batteries containing GPE of different ionic liquid content. Samples containing 40–50 vol % ionic liquid manifested superior stability and performance.

As presented in Figure 10, samples with ionic liquid exhibited an overall higher discharge capacity (~90 mAh/g) compared to the system with no ionic liquid. However, more specifically samples with 0 and >50 vol % ionic liquid exhibited poor stability and discharge capacity when samples with ionic liquid content between 25 and 50 vol % showed higher discharge capacity and stability. Low discharge capacity of 0 vol % samples is mainly due to the lack of ion conductivity. EC forms an ultra-thin film that acts as a protective layer, preventing damage to active materials on the electrodes. At higher concentrations (>50 vol %) of ionic liquid, this protective film is damaged over cycling and compromises the stability of the system, thus the average capacity of the system drops. At lower ionic liquid concentrations, this film remains intact, which results in maintenance of the system’s stability and performance. Average discharge capacities of the samples over 50 charging/discharging cycles are presented in Figure 10. Samples with 40–50 vol % ionic liquid show superior performance, which is in agreement with results from other reported experiments. Average rest voltages and discharge capacities are summarized in Table 4.

**Table 4:** The average rest voltages and discharge capacity of Li-ion polymer batteries as function of GPE composition.

Volume Percent of ILs (vol %)	Avg. Rest Voltage (V)	Avg. Discharge Capacity (mAh/g)
**0**	3.342	73.04
**25**	3.800	86.74
**30**	3.853	85.47
**40**	3.807	87.61
**50**	3.942	91.79
**60**	3.630	76.03
**75**	3.202	78.48
**100**	3.073	75.71

## 4. Conclusions

GPEs were characterized for their electrochemical properties. Chemical composition of GPEs was altered by the addition of ionic liquid at different volume ratios, and an optimum system was identified. GPEs consisting of 40–50 vol % EMI-Tf ionic liquid, in a 1:1 EC-PC solution exhibited highest performance, with considerably lower solution and charge transfer resistances, as well as highest rest voltage and discharge capacity.
